# Epigenetic Regulation in the Pathogenesis of Rheumatoid Arthritis

**DOI:** 10.3389/fimmu.2022.859400

**Published:** 2022-03-24

**Authors:** Chao Yang, Dan Li, Dehong Teng, Yueru Zhou, Lei Zhang, Zhangfeng Zhong, Guan-Jun Yang

**Affiliations:** ^1^ National Engineering Research Center for Marine Aquaculture, Institute of Innovation & Application, Zhejiang Ocean University, Zhoushan, China; ^2^ State Key Laboratory of Southwestern Chinese Medicine Resources, School of Pharmacy, Chengdu University of Traditional Chinese Medicine, Chengdu, China; ^3^ Department of Chemical Engineering, Waterloo Institute for Nanotechnology, University of Waterloo, Waterloo, ON, Canada; ^4^ Macau Centre for Research and Development in Chinese Medicine, Institute of Chinese Medical Sciences, University of Macau, Taipa, Macao SAR, China; ^5^ Key Laboratory of Applied Marine Biotechnology of Ministry of Education, Ningbo University, Ningbo, China; ^6^ State Key Laboratory for Managing Biotic and Chemical Threats to the Quality and Safety of Agro-products, Ningbo University, Ningbo, China; ^7^ Laboratory of Biochemistry and Molecular Biology, School of Marine Sciences, Ningbo University, Ningbo, China

**Keywords:** DNA methylation, histone modification, acetylation, inflammation, traditional chinese medicine

## Abstract

Rheumatoid arthritis (RA) is an autoimmune disease. The etiology of RA remains undetermined and the pathogenesis is complex. There remains a paucity of ideal therapeutic drugs and treatment strategies. The epigenetic modifications affect and regulate the function and characteristics of genes through mechanisms, including DNA methylation, histone modification, chromosome remodeling, and RNAi, thereby exerting a significant impact on the living state of the body. Recently, the phenomenon of epigenetic modification in RA has garnered growing research interest. The application of epigenetically modified methods is the frontier field in the research of RA pathogenesis. This review highlights the research on the pathogenesis of RA based on epigenetic modification in the recent five years, thereby suggesting new methods and strategies for the diagnosis and treatment of RA.

## Introduction

Rheumatoid Arthritis (RA) is a common autoimmune disease with certain specific pathological features, including joint deformities and dysfunction caused by chronic inflammation of the polyarticular synovium, formation of pannus, destruction of cartilage, and the subchondral bone (1, 2). Approximately 1% of people, worldwide, are affected by RA, a frequent cause for the loss and disability of the adult labor force. Currently, the pathogenesis of RA, which is mainly related to immune disorders, remains unclear (3). The morphological and gene expression patterns of RA synovial fibroblasts (RASF) are different from those of the normal synovial fibroblasts, are the key factors for the development of RA (4). In the absence of cellular and humoral immunity, RASF maintains its activated phenotype and destroys the cartilage, but its morphology and gene expression pattern are different from that of normal synovial fibroblasts, thus indicating that epigenetic modification plays a crucial role in RA pathogenesis (5). Based on the positivity of anticitrullinated antibodies, RA can be classified into two categories, ACPA+ and ACPA- (6). Genome-wide association analysis confirms that genetic variations are associated with RA incidence in different populations. However, these mutations only explain RA susceptibility in a small proportion of the ACPA+ population; the situation is much worse for the ACPA- RA population. Although smoking is the most vital environmental risk factor in the pathogenesis of RA, the relationship of immunity, lifestyle, genetic and environmental factors with their specific roles in the pathogenesis of RA remain unclear. Thus, epigenetic factors may be an important connecting link for genetics and gene expression, thereby facilitating the understanding of the pathogenesis of RA (7, 8).

The term “epigenetics” was first proposed by a British scientist, Waddington, in 1942. It refers to changes in the expressions and functions of genes resulting in heritable phenotypes even when the DNA sequences remain unchanged (9). There are three kinds of epigenetic modifications: (1) elective transcription and expression regulation of genes, such as through DNA methylation, chromatin remodeling, and genomic imprinting; (2) post-translational modifications of proteins, including histone methylation and acetylation; chemical or other modifications of histones, and covalent modifications of non-histone proteins; (3) post-transcriptional regulation of genes, including non-coding RNA, miRNAs, antisense RNAs, introns, and riboswitches in the genome (10). Epigenetics plays a significant role in autoimmune diseases including RA, as many studies show (11). This review summarizes the recent findings, in the last five years, on the roles and regulatory mechanisms underlying different epigenetic modifications in the development of RA, which may offer a new scientific perspective for the diagnosis and therapy of RA.

## The Epigenetic Regulatory Roles in RA

### DNA Methylation

DNA methylation is the most commonly occurring post-replication DNA modification in mammals, and consequently, is also one of the most extensively investigated epigenetic modifications (12). Under the action of DNA methyltransferases, the methyl group is covalently embedded on the fifth carbon atom of cytosine to form 5-methylcytosine (5-mc) (13). Generally, DNA methylation occurs in CpG islands in the promoter regions of housekeeping genes, wherein the guanine dinucleotides (CpG) are highly aggregated (14, 15). Hypermethylation in the promoter region is related to gene silencing or gene inactivation, while its hypomethylation activates transcriptional activity and promotes gene expression (16). According to the differences in their structures and functions, the methyltransferases were classified into three categories, namely, DNMT1, DNMT3a, and DNMT3b (17). DNMT1 is primarily responsible for maintaining the methylation status and is necessary for the *de novo* methylation of non-CpG sites (18). DNMT3a and DNMT3b exert important effects during embryonic development, and DNMT3b possesses a high density of CpG sites and can methylate the distal centromeric sites (19).

Previous studies show that the significantly altered feature of DNA methylation in synovial fibroblasts and peripheral blood mononuclear cells (PBMCs) of RA patients is the extensively hypomethylated genomic DNA. For example, the hypomethylation of the GC rich CpG island sequences on the promoter of the DNA of the long interspersed nuclear element in RASF affects the physiological and pathological processes, including adhesion plaque formation, cell adhesion, cross endothelial migration, and interactions with the extracellular matrix, thereby participating in the processes of the whole body or local joint inflammation in RA. All DNA hypomethylations in RASF are caused by the increase in the polyamine metabolism and a concomitant decrease in the levels of s-adenosine-l-methionine; not only do the patterns of DNA methylation change in RA but the promoter region of a single gene also undergo methylation, including those of the chemokine (CXC motif), ligand 12. The promoter demethylation of *IL-6* and *IL-10* genes in a single CpG sequence contribute to the increase in cytokine levels as the disease progresses. In addition, DNA methylation on chromosome 10 promotes the activation of fibroblast-like synovial cells (FLS) in RA pathogenesis (20). Inhibitors of DNA methylation suppress the release of cytokines and chemokines, as also the activation of FLS, thereby reducing the paw swelling. A recent study reports that the secreted frizzled-related protein 2 (SFRP2) is significantly downregulated in rats with RA (21). Over-expression of SFRP2 inhibits RA pathogenesis and suppressed the canonical Wnt signaling in fibroblast-like synovial cells (FLS) of RA rats (21). Interestingly, the level of expression of DNMT1 in RA rats is negatively correlated with that of SFRP2. Quantitative methylation-specific PCR confirms direct methylation of the SFRP2 promotor by DNMT1, thereby regulating FLS proliferation and fibronectin expression in a rat RA model. Therefore, the combination of DNMT1 and DNA methylation may be a promising therapeutic strategy for RA patients with down-regulated SFRP2 expression.

To investigate the epigenetic patterns of T lymphocytes in RA synovium, Firestein et al. analyzed the DNA sequence methylation patterns of CD3+ T cells in peripheral blood and synovial tissue from patients with RA and osteoarthritis (OA) (22). The differential sites of DNA methylation identified for RA and OA in the CD3+ T cells were 4615 and 164, respectively, while differentially methylated genes were 832 and 36, respectively. Further analyses showed that the differences in T-cell methylation were mainly related to their distribution (blood and synovium), and the differential modification pathways between RA blood and synovial T cells were mainly involved in complement activation, integrin cell surface interaction, and the p53 signaling pathway. Therefore, the specific immune characteristics of RA joints may be caused by the selective accumulation of the T-cell populations or the expansion of differentially labeled adaptive immune cells. Bernatsky et al. analyzed the differences in DNA methylation patterns between ACPA+ and ACPA- subjects and found 402 differentially methylated regions (DMRs) exerting genetic influence. These DMR-related genes were mainly enriched in pathways related to the Epstein-Barr virus infection and immune responses (23).

**Figure 1:**
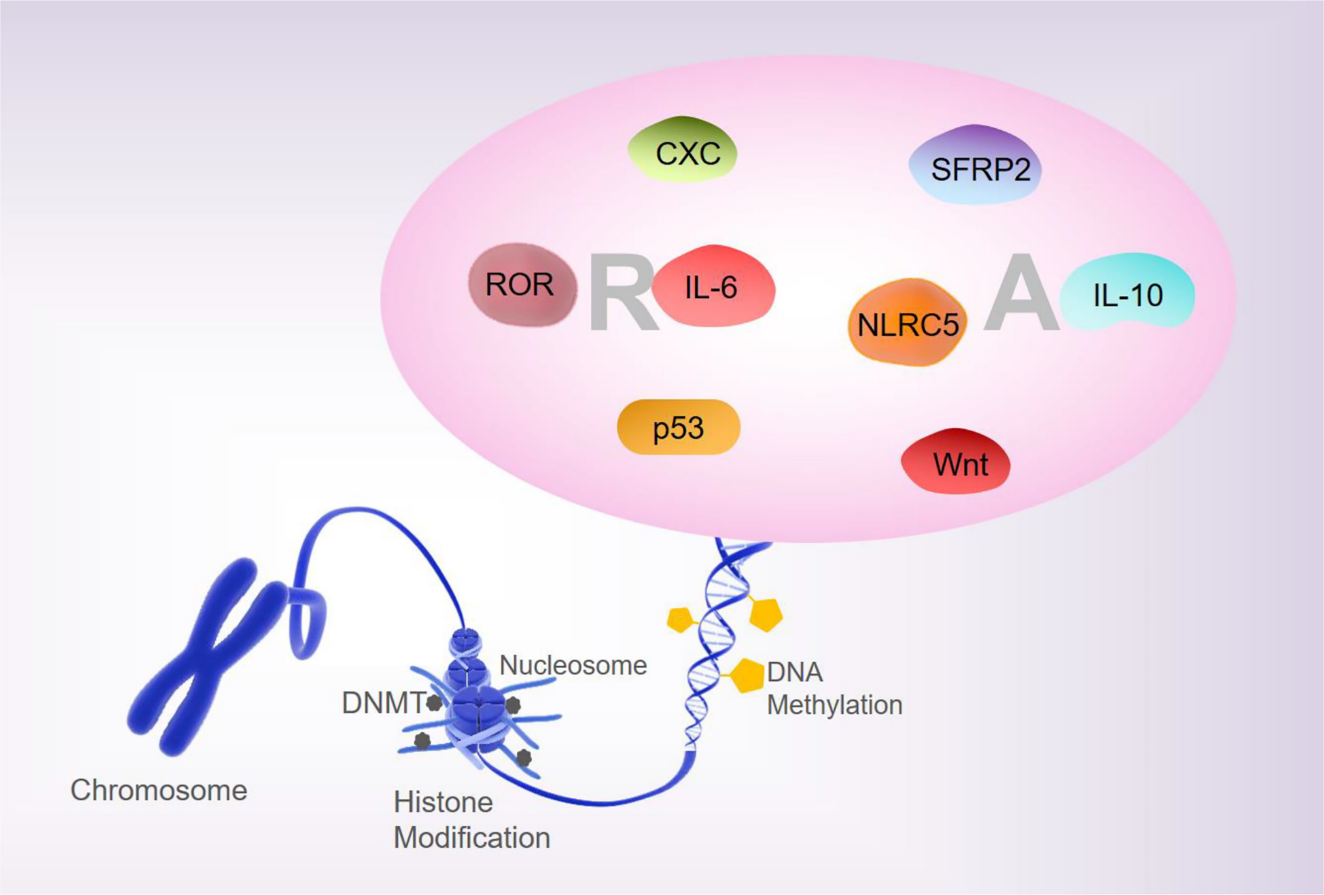
DNA methylation and RA. Changes in the methylation status of RA-related are involved in regulating the pathogenesis and progression of RA.

Analyses of the whole-genome DNA methylation and mRNA expression profiles of PBMCs from patients with RA show that approximately 1,046 DNA methylation sites are closely associated with the pathogenesis of RA (24). Liebold et al., using 5’-mC based flow cytometry, report that the PBMCs from RA patients show a significant overall DNA hypomethylation state relative to healthy people (25). The global methylation pattern may be a promising biomarker for therapeutic monitoring and prediction of the outcomes of inflammatory diseases. In addition, there are differences in the DNA methylation status in the B- and T- lymphocyte populations of patients with RA (26). Most differentially methylated positions (DMPs) in RA patients at the initial stages after treatment with disease-modifying antirheumatic drugs show increased DNA methylation, while most DMPs in RA patients at remission stages of MTX treatment showed reduced DNA methylation (27, 28). Therefore, the differential DNA methylation patterns are closely associated with the pathological processes of RA and can be used as the candidate biomarkers for evaluating the first-line drug responses in RA. The unbalance between T-regulatory (Treg)/T-helper (Th) 17 is engaged in the development of epigenetically-mediated autoimmune diseases. Relative hypermethylation of T-reg-specific demethylation region gene level and the hypomethylation of the retinoic acid-related orphan receptor (ROR)–C, have been reported to be detected in the early stages of active RA. Treg/Th17 imbalance is associated with stage, and aberrant patterns of DNA methylation may contribute to the pathogenesis of RA (29). Taken together, these findings suggest that altered methylation of RA factors is involved in the pathogenesis and progression of RA (**Figure 1**).

### Histone Modification

**Figure 2:**
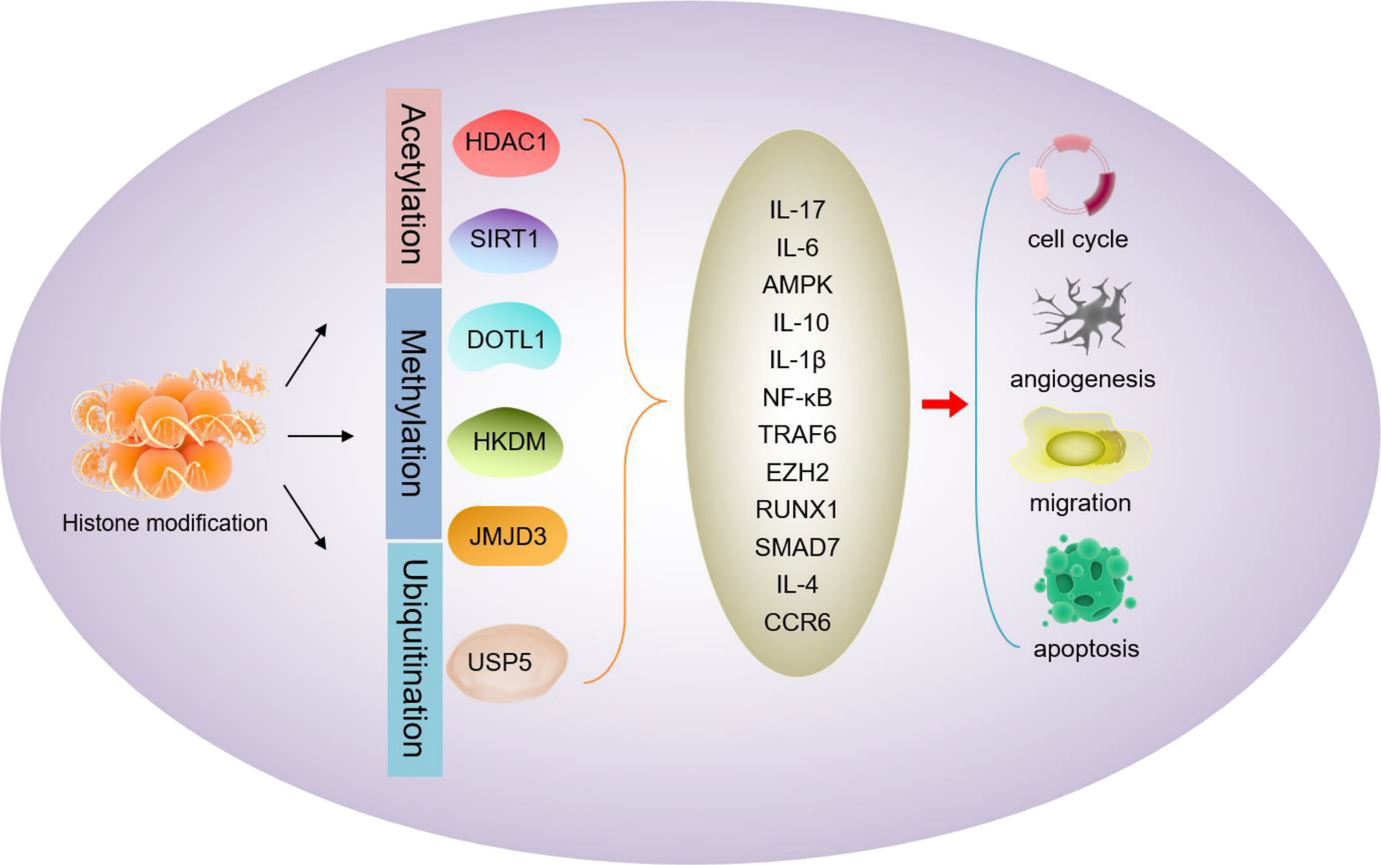
Schematic diagram of histone modifications mediating RA progression. Acetylation (HDAC1, SIRT1), methylation (DOTL1, HKDM, JMJD3), or ubiquitination (USP5) of RA-related proteins mediates the expression of RA factors (IL-17, IL-6, AMPK, IL-10, IL-1β, NF-κB, TRAF6, EZH2, RUNX1, SMAD7 IL-4, CCR6, etc.), thereby affecting the proliferation, invasion, and apoptosis of RA-related cells.

Histone modification is a post-translational modification of a specific site on histones in chromatin. Acetylation, methylation, phosphorylation, and ubiquitination are all included within the modifications of the histone tails; among which acetylation is the most common (**Figure 2**). HDACs are considered to play a significant role in the activation or silent regulation of pro-inflammatory genes, and their inhibitors are often used to study the pathogenesis of RA.

The expression and activity of class I HDACs are found to decrease in PBMCs of RA patients, which are implicated in disturbing the balance between the activities of HDAC and HATs (30). The hyperacetylation state caused by the decreased activity and expression of HDACs promotes the pro-inflammatory processes and ultimately leads to RA. Therefore, the HDAC activity and histone H3 acetylation status in PBMCs, are potential biomarkers for evaluating the disease activity. Mice with a T cell-specific deficiency of HDAC1 (HDAC1-cKO) were found to be resistant to the development of collagen-induced arthritis (CIA) and unaltered antibody response to type II collagen (31). Inflammatory cytokines, IL-17 and IL-6, were significantly reduced in the serum of HDAC1-cKO mice. Under the condition of high Th17, HDAC selective inhibitors inhibited chemokine receptor 6 (CCR6) upregulation in a mouse model and human CD4+ T-cells. Therefore, HDAC1 is not only a key factor in the pathogenesis of CIA but also a promising target for the treatment of RA patients.

SIRT1, is a type 3 histone deacetylase that, possesses anti-inflammatory properties. Activated SIRT1 promotes the phosphorylation of adenosine monophosphate-activated protein kinase α(AMPKα)/acetyl-CoA carboxylase in macrophages treated with interleukin IL-4, thereby upregulating the M2 genes, such as *MDC*, *FcϵRII*, *MrC1*, and *IL-10* expression (32). Moreover, activated SIRT1 downregulates the LPS/γ interferon-mediated NF-κB activity by inhibiting p65 acetylation and M1 genes (including *CCL2*, *iNOS*, *IL-12p35*, and *IL-12p40*) expression. This indicates that SIRT1 can reduce the inflammatory responses in RA by regulating M1/M2 macrophages polarization; thereby, SIRT1 is a potential target for the treatment of RA. In previous studies, high transcript and protein levels of DOT1L were detected in the synovial tissues of RA patients. The results of immunohistochemistry and western blotting proved a 13.8-fold or 15.5-fold increase in methylation of H3K79 in synovial tissue of RA patients, respectively (33). The role of DOT1L and H3K79 in initiating and maintaining gnomically active transcription important functions, indirectly demonstrating that histone modifications contribute to the pathogenesis of RA.

Abnormal histone lysine methylation (HKM) in RASF indicates that histone lysine methyltransferase (HKMT) and demethylase (HKDM) are dysregulated in RASF. Upon TNFα stimulation, the expressions of four HKDMs, including FBXL10, NO66, JMJD2D, and FBXL11, catalyzing the methylations of H3K4, H3K9, or H3K36 in RASF is higher than that of OASF. Therefore, the HKM modifying enzyme participates in altering HKM, leading to the changes in RASF gene expressions, thereby affecting the processes of RA (34). The JumonjiC histone demethylase (JMJD3) family is implicated in the regulation of the FLS proliferation and activation, which is associated with joint destruction and pathological processes in RA (35). JMJD3 expression is significantly upregulated in RA-FLS; in FLS, these enhanced levels are induced by the platelet-derived growth factor (PDGF), as it promotes proliferation and migration of FLS. Inhibiting the activity of JMJD3 significantly reduces FLS proliferation and migration. Knockdown of JMJD3 eliminates PDGF-induced PCNA expression in FLS; additionally, it reduces the inflammatory responses of SFs treated with IL-1β (36). Therefore, JMJD3 plays an essential role in the development of RA. Hence, targeting JMJD3 may serve as a new strategy for the diagnosis and treatment of RA.

Ubiquitination is an essential protein post-translational modification mechanism whose main role includes protein degradation and its functional regulation. The expression of ubiquitin-specific protease 5 (USP5) is significantly upregulated upon stimulation with IL-1β which increases the USP5 levels in a time-dependent manner in RA-FLS (37). Overexpression of USP5 significantly stimulates the production of pro-inflammatory cytokines and activation of the related nuclear factor kappa B (NF-κB) signaling pathway. USP5 interacts with the tumor necrosis factor receptor-related factor 6 (TRAF6), an E3 ubiquitin ligase, and a key cytoplasmic signaling adaptor involved in the regulation of key biological processes. Thus, USP5 inhibits polyubiquitination and stabilizes TRAF6. Inhibition of TRAF6 can reduce collagen-induced bone loss and MMP expression in rats with RA; thus, TRAF6 may be an alternative treatment target for RA (38). Low levels of chromatin modifier zeste homolog 2 (EZH2) expression were detected in PBMCs and CD4+ T-cells from RA patients, which may be attributed to the partial neutralization of EZH2 expression by anti-IL17 antibodies. The downregulation of the EZH2 activity suppresses the differentiation of the Tregs and transcription FOXP3. Moreover, it downregulates the RUNX1, while upregulating the expression of SMAD7 in CD4+ T cells (39). In addition, aberrations of EZH2 in CD4+ T-cells may contribute to the lack of Tregs in RA patients.

Long non-coding RNA maternally expressed gene 3 (MEG3) is a tumor suppressor that is also imprinted and involved in the occurrence of several tumors. Li et al. report that MEG3 levels are down-regulated in synovial tissues and FLS in a complete Freund’s adjuvant (CFA) induced rat RA model. Overexpression of MEG3 decreased the levels of NLRC5 and inflammatory cytokines. The data for methylation-specific PCR suggest that the MEG3 gene promoter is significantly methylated in the CFA-induced synovial tissue and FLS. However, DNA methyltransferase 1 (DNMT1) is reported to be significantly upregulated in the CFA-induced synovial tissues and cells, suggesting that methylation inhibitors can attenuate the hypermethylation of the MEG3 promoter. Therefore, the imprinted gene MEG3 affects the processes of RA by targeting NLRC5 in an effect to regulate the levels of methylation (40).

### MicroRNA-Mediated Pathogenesis of RA

miRNAs are endogenous non-coding single-stranded small RNAs of about 22 nucleotides in length. These are widely present in all organisms. Binding to the 3’-untranslated region of the target genes, miRNAs act as a post-translational repressor of the gene leading to its degradation or translational repression. miRNAs are involved in regulating approximately 30% of all gene expression and translation patterns crucial for life processes of cellular proliferation, differentiation, metabolism, inflammation, and apoptosis. Recent studies show that miRNAs participate in epigenetic regulation by regulating the levels of DNA methylation or changing histone modifications, which in turn affect the onset and progression of RA (**Table 1**). The miRNA-targets are repeatable in Grand (https://grand.networkmedicine.org/). A glossary of genes and form symbols is shown in **Table 2**.

**Table 1:** Representative miRNAs associated with RA pathogenesis and progression.

miRNAs	Expression	Source	Type	Effect	Reference
*miR-124*	upregulation	RA patients	synovial tissues	Inhibit cell proliferation promote apoptosis	(41)
*miR-126*	upregulation	RA patients	Serum, synovial tissue and synovial fluid	Inhibit apoptosis promote proliferation	(42)
*miR-128-3p*	upregulation	RA patients	PBMC	Regulate the activity of inflammatory factors	(43)
*miR-138*	upregulation	RA patients	synovial tissues	Regulate the activity of inflammatory factors	(44)
*miR-145-5p*	downregulation	RA patients	synovial tissues	Promote cell proliferation and inflammatory factor expression	(45)
*miR-155-5p*	downregulation	RA patients	plasma	up-regulation of pro-inflammatory cytokines	(46)
*miR-17*	downregulation	RA patients	synovial tissues	Anti-inflammatory and anti-erosion	(47)
*miR-410-3p*	downregulation	RA patients	synovial tissues	Inhibit cell proliferation promote apoptosis	(48)
*miR-21*	upregulation	RA patients	synovial tissues	Inhibit cell invasion and inflammatory factor expression	(49)
*miR-221-3p*	upregulation	RA patients	PBMC	Promote inflammatory factor expression	(50)
*miR-23b*	upregulation	RA patients	fibroblast-like synoviocytes	Inhibit inflammatory cytokine expression	(51)
*miRNA-340-5p*	downregulation	RA patients	serums, synovial tissues, FLSs	Inhibit cell proliferation promote apoptosis	(52)
*miR-431-5p*	downregulation	RA patients	synovial tissues, FLSs	Inhibit cell proliferation promote apoptosis	(53)
*miR-449*	downregulation	RA patients	synovial tissues	Inhibit cell proliferation promote apoptosis	(54)
*miR-4701-5p*	downregulation	RA patients	FLSs	Inhibit cell proliferation promote apoptosis	(55)
*miR-574-5p*	upregulation	RA patients	synovial fluid	promote bone destruction	(56)
*miR-590-5p*	downregulation	RA patients	synovial tissues, FLSs	Inhibit cell proliferation and invasion	(57)
*miR-6089*	downregulation	RA patients	FLSs, synovial tissues	Inhibit cell proliferation induce apoptosis	(58)
*miR-613*	downregulation	RA patients	Synovial tissues	Inhibit cell proliferation and invasion promote apoptosis	(59)
*miR-650*	downregulation	RA patients	Synovial tissues	Inhibit cell proliferation and invasion promote apoptosis	(60)

**Table 2:** The glossary of all genes and formal symbols is mentioned in this paper.

Genes	Formal symbols	Definitions
*DNMT1*	DNMT1	DNA (Cytosine-5-)-Methyltransferase 1
*DNMT3a*	DNMT3A	DNA (Cytosine-5-)-Methyltransferase 3 Alpha
*DNMT3b*	DNMT3B	DNA (Cytosine-5-)-Methyltransferase 3 Beta
*SFRP2*	SFRP2	Secreted Frizzled Related Protein 2
*TP53*	TP53	Tumor Protein P53
*RORC*	RORC	RAR Related Orphan Receptor C
*HDAC1*	HDAC1	Histone Deacetylase 1
*IL-6*	IL-6	Interleukin 6
*IL-17*	IL-17	Interleukin 17
*CCR6*	CCR6	C-C Motif Chemokine Receptor 6
*IL-1β*	IL1B	Interleukin 1 Beta
*TNF-α*	TNF	Tumor Necrosis Factor
*VEGF*	VEGFA	Vascular Endothelial Growth Factor A
*EGF*	EGF	Epidermal Growth Factor
*JUN*	C-Jun	Jun Proto-Oncogene, AP-1 Transcription Factor Subunit
*AGXT*	AGXT	Alanine-Glyoxylate And Serine-Pyruvate Aminotransferase
*RLP3*	RLP3	Ribosomal Protein L3
*AHCY*	AHCY	S-Adenosyl-L-Homocysteine Hydrolase
*NFKB1*	NFKB1	Nuclear Factor Kappa B Subunit 1
*PGE2*	PGE2	Prostaglandin E Receptor 2
*RANKL*	TNFSF11	TNF Superfamily Member 11 2
*PDK1*	PDK1	Pyruvate Dehydrogenase Kinase 1
*AKT*	AKT1	AKT Serine/Threonine Kinase 1
*TRAF6*	TRAF6	TNF Receptor Associated Factor 6
*Ubc13*	UBE2N	Ubiquitin Conjugating Enzyme E2 N
*HDAC6*	HDAC6	Histone Deacetylase 6
*IL-12*	IL-12	Interleukin 12
*IL-10*	IL-10	Interleukin 10
*MMP2*	MMP2	Matrix Metallopeptidase 2
*MMP9*	MMP9	Matrix Metallopeptidase 9
*PI3K*	PIK3CA	Phosphatidylinositol-4,5-Bisphosphate 3-Kinase Catalytic Subunit Alpha
*MMP1*	MMP1	Matrix Metallopeptidase 1
*MMP3*	MMP3	Matrix Metallopeptidase 3
*CCL2*	CCL2	C-C Motif Chemokine Ligand 2
*CXCL8*	CXCL8	C-X-C Motif Chemokine Ligand 8
*CXCL10*	CXCL10	C-X-C Motif Chemokine Ligand 10
*NR1D1*	NR1D1	Nuclear Receptor Subfamily 1 Group D Member 1
*AKT2*	AKT2	AKT Serine/Threonine Kinase 2
*DKK1*	DKK1	Dickkopf WNT Signaling Pathway Inhibitor 1
*MAP2K3*	MAP2K3	mitogen-activated protein kinase kinase 3
*STAT3*	STAT3	Signal Transducer And Activator Of Transcription 3
*JAK3*	JAK3	Janus Kinase 3
*CXCL13*	CXCL13	C-X-C Motif Chemokine Ligand 13
*TLR4*	TLR4	Toll Like Receptor 4
*FOXP3*	FOXP3	Forkhead Box P3
*JAK1*	JAK1	Janus Kinase 1
*IL-23R*	IL-23R	Interleukin 23 Receptor
*MARCKS*	MARCKS	Myristoylated Alanine Rich Protein Kinase C Substrate
*XIAP*	XIAP	X-Linked Inhibitor Of Apoptosis
*Notch*	Notch1	Notch Receptor 1
*NLRC5*	NLRC5	NLR Family CARD Domain Containing 5
*MEG3*	MEG3	Maternally Expressed 3
*SMAD7*	SMAD7	SMAD Family Member 7
*EZH2*	EZH2	Enhancer Of Zeste 2 Polycomb Repressive Complex 2 Subunit
*IL-8*	IL-8	Interleukin 8
*TRAF2*	TRAF2	TNF Receptor Associated Factor 2
*cIAP2*	BIRC3	Cellular Inhibitor Of Apoptosis 2
*PGRN*	GRN	Granulin Precursor
*HDAC4*	HDAC4	Histone Deacetylase 4
*GAS5*	GAS5	Growth arrest-specific transcript 5
*IFN-γ*	IFNG	Interferon Gamma
*PIK3R2*	PIK3R2	Phosphoinositide-3-Kinase Regulatory Subunit 2
*JMJD3*	KDM6B	JmjC Domain-Containing Protein 3
*USP5*	USP5	Ubiquitin-specific protease 5
*RUNX1*	RUNX1	RUNX Family Transcription Factor 1
*FBXL10*	KDM2B	F-Box And Leucine-Rich Repeat Protein 10
*NO66*	RIOX1	Ribosomal Oxygenase 1
*JMJD2D*	KDM4D	Jumonji Domain-Containing Protein 2D
*FBXL11*	KDM2A	F-Box And Leucine-Rich Repeat Protein 11
*DOT1L*	DOT1L	DOT1 Like Histone Lysine Methyltransferase
*SIRT1*	SIRT1	Sirtuin 1
*iNOS*	NOS2	Nitric Oxide Synthase 2
*CCL2*	CCL2	C-C Motif Chemokine Ligand 2
*MDC*	CCL22	C-C Motif Chemokine Ligand 22
*FcϵRII*	FCER2	Fc Epsilon Receptor II
*MrC1*	MRC1	Mannose Receptor C-Type 1
*AMPK α*	PRKAA1	Protein Kinase AMP-Activated Catalytic Subunit Alpha 1
*ACACA*	ACACA	Acetyl-CoA Carboxylase Alpha

HDAC1 was reported to be highly expressed in synovial tissues of CIA, while miR-124 and MARCKS were lowly expressed. Silencing or inhibiting HDAC1 can increase the expression of MARCKS and miR-124 by promoting the H3 and H4 acetylation of promoter regions of miR-124 and the MARCKS. miR-124 reduces the proliferation of synovial cells and inflammation of the synovium by inhibiting the JAK/STAT signaling pathway in CIA. Therefore, increasing the expressions of miR-124 and MARCKS by silencing HDAC1 to reduce synovial cell proliferation and synovial inflammation in CIA is a promising new strategy for RA treatment as evidenced from the results in the mouse model (41). In addition, miR-449 exerts a protective effect against RA by targeting HDAC1 to inhibit the proliferation of RASFs and induce their apoptosis (54).

The lentivirus, Lv-miR-126, significantly increases miR-126 expression in RASF, and simultaneously, promotes the proliferation of RASF and inhibits cellular apoptosis. In addition, decreased levels of PIK3R2 and increased levels of those of PI3K and p-AKT were detected in RASFs overexpressing miR-126. Co-transfection of anti-miR-126 and PIK3R2 siRNA constructs further increased PI3K and p-AKT levels while enhancing RASF proliferation and reducing apoptosis. The luciferase reporter gene assays indicate that miR-126 directly interacts with PIK3R2. In general, overexpression of miR-126 expresses PIK3R2 and apoptosis and promotes the proliferation of RASF (42). In another study, inhibition of miR-126 expression was found to significantly upregulate TNF-α, IFN-γ, and IL-23R levels in RA patients. Correspondingly, the overexpression of miR-126 in FLS enhanced the levels of IL-23R, TNF-α, and IFN-γ, indicating that miR-126 negatively regulates the expressions of IL-23R, TNF-α, and IFN-γ, thereby affecting the processes of RA (42).

Jiang et al. show that long-chain non-coding RNA growth arrest-specific transcript 5 (GAS5) can ameliorate RA progression by inducing apoptosis in RA-FLS. Further mechanistic examination showed that the overexpression of miR-128-3p or HDAC4 knockdown attenuated the inhibitory effects of GAS5 or anti-miR-128-3p on the development of RA. GAS5, a miR-128-3p sponge, upregulates the expression of HDAC4. Thus, GAS5 partially regulates HDAC4 through miR-128-3p to inhibit inflammation in synovial tissues (44). miR-138, which is highly expressed in the serum and synovial tissues of RA patients, negatively regulates HDAC4 to mediates the activities of NF-κB, PGRN, and RA-related inflammatory cytokines in an acetylation-dependent manner. High expression of miR-138 in the serum and synovial tissues of RA patients negatively regulates HDAC4, which in turn regulates NF-κB and PGRN in an acetylation-dependent manner, thereby affecting the RA-related inflammatory cells and RA factors (61).

miR-145-5p is the target miRNA of the long non-coding RNA, PVT1. The expressions of PVT1 and miR-145-5p are negatively correlated in the synovial tissues of RA patients; thus, the expression of miR-145-5p was reduced, whereas the expression of PVT1 increases significantly. In addition, the tumor necrosis factor-α (TNF-α) stimulates an increase in PVT1 in RA-FLS and suppresses the level of miR-145-5p. Knockdown of PVT1 inhibits TNF-α-induced over-proliferation of RA-FLS, suppressed interleukin (IL)-1β, and IL-6 production, and suppressed NF-κB activation mediated by miR-145-5p. The above findings show that PVT1 regulates the apoptotic and inflammatory responses of RA-FLS by targeting miR-145-5p (45).

Ahmed et al. show that miR-17 negatively regulates TNF-α by mediating the protein ubiquitination processes in RASF. miR-17 increases the polyubiquitination of K48-linked TRAF2, cIAP1, and cIAP2 stimulated by TNF-α in RASF. Therefore, the destruction of TRAF2 by miR-17 reduces the ability of TRAF2 to bind to cIAP2, thereby reducing TNF-α-induced nuclear translocation of NF-κBp65, c-Jun, and STAT3, and production of IL-6, IL-8, MMP-1, and MMP-13 in RASF (62). In addition, miR-17 inhibits the proteins of the IL-6 family by directly targeting JAK1 and STAT3 signaling cascades, thereby exerting anti-inflammatory and anti-erosion effects. MicroRNA-17 directly targets the 3’-untranslated regions of STAT3 and JAK1, resulting in a decrease in the mRNA and protein expressions of STAT3 and JAK1 (47).

In response to TNF-α stimulation, the expression of long non-coding RNA NEAT1 is upregulated in the synovial tissues and RA-FLS. It promotes cell proliferation and inflammatory cytokines secretion. NEAT1 directly binds and negatively regulates the miR-410-3p expression (48). Inhibition of miR-410-3p can partially rescue the inhibitory effects on cell viability induced by NEAT1 depletion in RA-FLS. Knocking out NEAT1 attenuates the TNF-α-induced proliferation and inflammatory cytokines production in RA-FLS; simultaneously, it promotes cellular apoptosis through miR-204-5p (63). These findings suggest that NEAT1 may serve as a sponge of multiple miRNAs and is a potential treatment target for RA.

Treg transcription factor FoxP3 shows high expression in inactive RA and repression in active RA, while the Th17 transcription factor RORc shows the opposite trend, as shown in some previous studies (64). The upregulation of miR-21 increases the proportion of Tregs and decreases that of the Th17 cells, thereby regulating the Treg/Th17 balance and resulting in favorable progression of RA (65). In addition, high levels of miR-21 can inhibit the expressions of IL-6 and IL-8 by suppressing the Wnt signaling pathway, thereby alleviating the symptoms of RA (66).

Evidence suggests that macrophages with an inflammatory phenotype (M1) predominate in the synovium of RA relative to those with the anti-inflammatory phenotype (M2). Regardless of RA patients or healthy individuals, the expressions of miR-221-3p and miR-155-5p in M1 are significantly higher relative to the M2 macrophages. miR-221-3p promotes IL-6 and IL-8 secretion in M2-macrophages but suppresses those of IL-10, CXCL13, JAK3, and the activation of pSTAT3. JAK3, a target of miR-221-3p, is involved in mediating the functions of the inflammatory M2-macrophages induced by TLR4 (50).

Wang et al. report that plasma levels of miR-23b are significantly higher in RA patients relative to healthy controls. Meanwhile, plasma miR-23b expression is significantly related with levels of hemoglobin (Hb), total bilirubin (TBIL), direct bilirubin (DBIL), indirect bilirubin (IBIL), total cholesterol (TC), and low-density lipoprotein cholesterol (LDL-C level is negatively correlated) (P <0.05). After receiving appropriate treatments, the plasma miR-23b level decreased in RA patients. Therefore, circulating miR-23b may be a promising biomarker for evaluating RA disease activity (51).

The expression of miRNA-340-5p is significantly downregulated in the serum, synovial tissue, and RA-FLS of patients with RA. The overexpression significantly inhibited cell proliferation and inflammatory factor expression in RA-FLS and promotes apoptosis (52). Similarly, miR-431-5p is down-regulated in the synovial tissue and FLS of RA patients. Overexpressed miR-431-5p can inhibit the proliferation of cells and promote cellular apoptosis, indicating its prospect in the treatment of RA (53).

Long non-coding RNAs play a key role in several autoimmune diseases, including rheumatoid arthritis (RA). Zheng et al. identified a long intergenic non-protein-coding RNA162 (LINC00162, also known as lncRNA PICSAR: p38 inhibited cutaneous squamous cell carcinoma associated lincRNA), which is highly expressed in RA-FLS and RA synovial fluid (55). Inhibition of LINC00162 significantly alters the proliferation, migration, invasion, and production of pro-inflammatory cytokines from RA-FLS cells. Further studies show that miR-4701-5p acts as a sponge of LINC00162 and regulates its function. Therefore, miR-4701-5p may be a potential treatment target for RA.

Characterized by synovial inflammation and joint destruction, RA is a chronic autoimmune disease. Intercellular communication in the synovial microenvironment is mediated by small cell-derived extracellular vesicles (sEVs) as they carry microRNAs. Saul et al. show that sEVs in the synovial fluid of RA patients significantly promote osteoclast differentiation through miR-574-5p-mediated activation of TLR7/8. miR-574-5p is a key mediator in RA pathogenesis and may be a potential target in the fight against bone destruction (56).

Zhao et al. report that miR-590-5p alleviates RA by inhibiting the MAPK signaling pathway. miR-590-5p inhibits the expression of mitogen-activated protein kinase kinase 3 (MAP2K3) in RA-FLS post-transcriptionally, thereby inhibiting FLSs proliferation and invasion. Inhibitors of miR-590-5p or its sponge linc02381 enhance the MAP2K3 expression and activation of p38 and AP-1 in the MAPK signaling pathway, thereby aggravating the pathogenesis of RA. Therefore, upregulation or overexpression of miR-590-5p can alleviate the pathogenesis of RA (57).

Compared to the healthy controls, the expression of miR-6089 was significantly lower in the synovial tissue and FLS of RA patients. Upregulation or overexpression of miR-6089 in RA-FLS inhibits cell proliferation and induces apoptosis along with the expressions of cleaved-caspase-3, -8, and -9 proteins. In addition, AKT1 serves as a direct target of miR-6089. miR-6089 regulates inflammation through the AKT1/NF-κB signaling pathway (67). As a result, miR-6089 may be a promising target for the prevention and treatment of RA.

The Dickkopf Wnt Signaling Pathway Inhibitor 1 (DKK1) is the main regulator of joint remodeling. DKK1 is upregulated in RA tissues and RASF, thereby aggravating joint destruction. The significantly downregulated miR-613 in RA tissues and RASF can bind to and suppress the DKK1 expression. miR-613 or DKK1 knockdown inhibits the proliferation and invasion of RASF and induces its apoptosis. Hence, one of the mechanisms for alleviating or treating RA is to inhibit the proliferation and invasion of RASFs and induce apoptosis by regulating DKK1 expression (59).

In RASFs from 16 patients with rheumatoid arthritis (RA) and 13 patients with joint trauma who underwent joint replacement surgery, miR-650 was down-regulated, whereas AKT2 was up-regulated (60). Detection of the dual-luciferase reporter genes revealed that miR-650 is specifically bound to the 3’-untranslated region of AKT2, thereby downregulating AKT2 expression. Further downregulation of miR-650 or upregulation of AKT2 in RASF increases cell proliferation, migration, and invasion while decreasing the occurrence of apoptosis. Therefore, suppressing the expression of AKT2 in RASF may be the mechanism by which miR-650 may function as a potential therapeutic target for RA.

## The Effects of Compounds on Epigenetic Regulation of RA

### The Effect of Epigenetic Inhibitors on RA

Cho et al. show that the histone deacetylase (HDAC) inhibitor, suberoylanilide hydroxamic acid (SAHA), reduces the clinical score and the incidence in mice with collagen-induced arthritis (CIA). SAHA relieves CIA by specifically inhibiting Th17 cell differentiation and Th17 cell-related transcription factors expression through NR1D1. Therefore, the histone deacetylase (HDAC) inhibitor, SAHA, may be a potential therapeutic agent for RA (68). Similarly, Song et al. report that the overexpression of HDAC6 in macrophages results in the enhanced expressions of TNF-α and IL-6. Downregulation or the treatment with HDAC6 inhibitor, CKD-506, significantly reduces the TNF-α and IL-6 production from the PBMCs of activated RA patients. CKD-506 directly or indirectly inhibits the proliferation of Teffs by regulating the functions of iTregs. In addition, CKD-506 improves the clinical arthritis score of AIA rats in a dose-dependent fashion. Moreover, the combination of CKD-506 and methotrexate also produce a synergistic effect on RA (69). Recently, the anti-RA activity of M808, a selective inhibitor of HDAC6, has been evaluated. M808 down-regulates the production of IL-1β-related RA factors, including MMP-1, MMP-3, IL-6, CCL2, CXCL8, and CXCL10. M808 increases the clinical arthritis score of AIA mice in a dose-dependent style. Besides, M808-induced HDAC6 inhibition is implicated in milder synovial inflammation and joint destruction. Therefore, HDAC6 inhibitors may be used as potential therapeutic drugs for RA (70).

Choudhary et al. report that the HDAC1 inhibitor, phenethyl isothiocyanate (PEITC), possesses potential anti-RA activity. The preventive treatment using PEITC reduces paw edema, total arthritis index, mobility score, stair climbing ability, behavioral parameters, and bone erosion in rats with CFA-induced arthritis in a dose-dependent manner. In addition, PEITC expressively downregulates the level of TNF-α in the synovial tissues of the CFA rats (71).

Trichostatin A (TSA), an inhibitor of HDACs, also exhibits potential anti-RA activity. TSA significantly inhibits the proliferation, invasion, and apoptosis of RA-FLS under hypoxic conditions. Further mechanistic verification shows that the anti-RA activity of TSA is related to the inactivation of PI3K/Akt signaling evidenced by the suppression of the matrix metalloproteinases (MMP-2 and MMP-9) and PI3K expression, as well as the phosphorylation of Akt (72).

Since pan-HDACi inhibits all of the 11 Zn2+-dependent HDACs and causes a wide range of side effects, it was hypothesized that specific inhibitors of histone deacetylase 6 (HDAC6i) are would have fewer side effects. Therefore, Mahboobi et al. developed a new selective drug, Marbostat-100, that targets HDAC6. Marbostat-100 can effectively improve arthritis induced by type II collagen and shows good drug resistance (73).

The E3 ligase, TNF receptor-related factor 6 (TRAF6), is involved in chronic immune stimulation in several diseases, including autoimmune disorders, inflammation, and cancer. TRAF6 is highly expressed in patients with RA and SLE, and it interacts with Ubc13 to activate the NF-κB signaling pathway, thereby promoting the processes of RA. Brenke et al. developed the first inhibitor of TRAF6-Ubc13 protein-protein interaction, C25-140. C25-140 strongly inhibits the activation of NF-κB signaling in a variety of immune and inflammatory signaling pathways in both human and mouse primary cells. Notably, C25-140 has been shown to reduce inflammation in preclinical mouse models and improve the autoimmune RA status (74).

As mentioned above, GSK-J4, as an inhibitor of JMJD3, suppresses inflammatory responses by suppressing IL-1β-induced upregulation of TLR2 and COX-2 (36). Furthermore, GSK-J4 regulates osteoclastogenesis and differentiation. The number of TRAP+ multinucleated cells was significantly reduced in the presence of lower concentrations of GSK-J4, with little effect on cell viability. After continuous daily intraperitoneal injection of GSK-J4 (20 mg/kg body weight, n = 8) for 40 days, the severity of arthritis in CIA mice was significantly reduced.

### The Effects of Traditional Chinese Medicine-Mediated Epigenetic Regulation on RA

The classic traditional Chinese medicine (TCM), Wutou Decoction (WTD), has been clinically used for thousands of years and has proven to be reliably efficient and safe in the treatment of RA. WTD exerts anti-inflammatory effects by regulating DNA methylation and histone modification. The mRNA level expressions of DNMT1 and DNA methylation in CIA rats treated by WTD gavaging were found to be significantly downregulated. In addition, WDT increases the level of H3 acetylation in PBMCs (75). In-depth mechanistic studies show that the five herbs in WTD exert synergistic anti-arthritis effects in RA. Among them, aconite (AC) is the main anti-inflammatory active substance; the auxiliary component, ephedra (EP), can significantly inhibit the NF-κB-mediated inflammatory responses. Another auxiliary ingredient, astragalus (AS), whether used alone or in combination with AC, significantly upregulates the expression of Nrf2. Nevertheless, WTD is better than any combination of ingredients in the treatment of RA (76).

Tripterygium wilfordii is extensively used in TCM for the treatment of autoimmune diseases, such as RA and systemic lupus erythematosus. The structure-optimized analog of its extract, (5R)-5-hydroxy triptolide (LLDT-8), has high immunosuppressive and low toxicity capacities. Notably, LLDT-8 also inhibits the differentiation of Th1 and Th17 cells, thereby impacting the immune responses in RA patients. He et al. showed that significant differential expression of 394 genes (281 downregulated and 113 upregulated) was found in FLS under LLDT-8 treatment. KEGG enrichment analysis indicated that 20 pathways associated with immune response were significantly enriched, including cytokine-cytokine receptor interaction (P=4.61×10^-13^), chemokine signaling pathway (P=1.01×10^-5^), and the TNF signaling pathway (P = 2.79 × 10^-4^) (77).

The main active substance isolated from the oldest and most commonly used Chinese medicine, astragalus, is astragalus total flavonoids (TFA), which increases FCA-induced weight in rats, reduces primary foot swelling, and arthritis index, as well as the thymus and spleen indexes. Mechanistic studies show that TFA inhibits the production of TNF-α, IL-1β, PGE2, and RANKL, and promotes that of OPG in rat serum induced by FCA. These results show that the OPG/NF-κB pathway is one of the main mechanisms underlying the effects of astragalus against FCA-induced RA (78). In addition, previous studies report that astragaloside IV (AST) increases miR-17-5p expression in FLS, and downregulates those of lncRNA LOC100912373, PDK1, and p-AKT in an effect to inhibit cell proliferation (79).

Xu et al. explored the mechanism of action of the Baihu Guizhi Decoction in a model of histopathological heat joint pain (PA). The Baihu Guizhi Decoction improves foot swelling and pathological damage and significantly inhibits the expressions of IL-1β, TNF-α, EGF, VEGF, IL-17, and IL-12p70 in the PA model. In addition, the mRNA levels of methylated genes *AHCY*, *RPL3* were down-regulated, while the mRNA levels of *Agxt* were up-regulated. Therefore, Baihu Guizhi Decoction can alleviate RA by regulating the unique synovial gene methylation pattern in the PA model (80).

## Conclusion and Outlook

**Figure 3:**
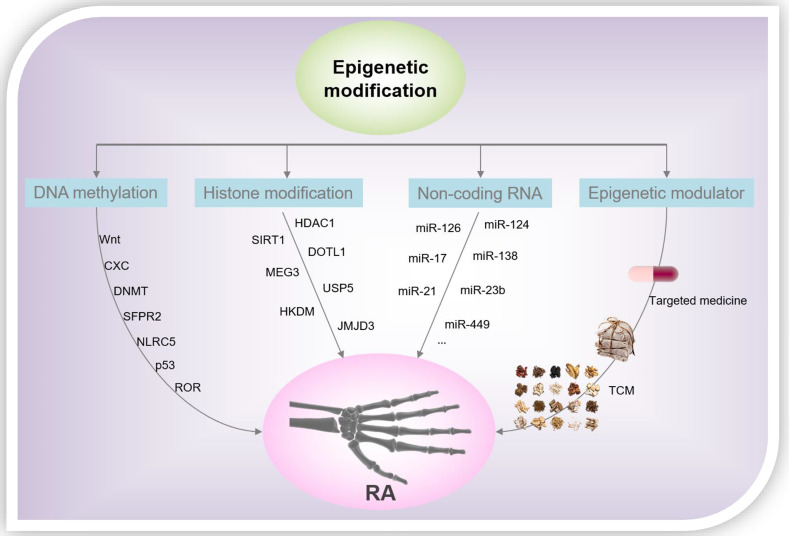
RA-related epigenetic modification and drug intervention.

From the foregoing discussion, it is clear that epigenetics plays a significant regulatory role in the processes of gene transcription and is participate of the onset and progression of several diseases. Epigenetics is closely related to the pathological mechanism underlying RA, and we summarized the representative DNA methylation, histone modification patterns, and miRNAs in RASF over the past 5 years (**Figure 3**). However, the study of epigenetics in the pathogenesis of RA is still in its infancy, and more research is needed to further analyze its roles underlying the progression of RA. Although pan-hypomethylation in RA has been demonstrated, key pathogenic and therapeutic proteins, as well as genes, need to be identified. Histone acetylation inhibitors, in particular the HDAC1 antagonists, show good anti-inflammatory effects in the laboratory, and more experiments are still needed to verify their therapeutic effects in clinical settings. In addition, investigations to verify the role of other modifications of histones, such as ubiquitination and phosphorylation, in RA, are potential research directions. On the other hand, the toxicity/side effects of rheumatoid arthritis drugs due to the induction of epigenetics should be concerned. For example, the neurotoxicity of methotrexate is caused by changes in epigenetic modifications that it induces during myelination (81). Epigenetic changes that miRNA-mediated is directly implicated in the aberrant expression of RA-related genes and ultimately determine the reversibility of cellular functions and pharmacology. TCM plays a vital role in the health of the Chinese people and even human beings, especially in the global fight against COVID-19 (82–85). However, due to the complex components of TCM, the epigenetic modification of RA caused by TCM is a comprehensive effect of multiple pathways, multiple targets, and multiple mechanisms. Therefore, it is necessary to create more effective research methods to clarify the mechanism of action of TCM in the treatment of RA, and to provide theoretical and data references for the modernization of TCM. An in-depth understanding of the epigenetic regulatory mechanisms of RA will help identify new markers, signaling pathways, and target drugs/TCM, leading to new strategies for the diagnosis and treatment of RA.

## Author Contributions

CY and G-JY conceived and designed the whole project. CY, DL, and ZZ drafted the manuscript. DT, YZ, and LZ revised the manuscript. All authors reviewed and approved the final manuscript.

## Funding

The work was supported by the General Scientific Research Project of Education of Zhejiang Province (Y202147351), Multidisciplinary interdisciplinary innovation team for multidimensional evaluation of southwestern characteristic Chinese medicine resources (NO. ZYYCXTD-D-202209), the Starting Research Fund of Ningbo University (421912073), National Natural Science Foundation of China (82104477, U19A2010, and 81891012), special support from China Postdoctoral Science Foundation (2019M663456 and 2019TQ0044), Xinglin Scholar Research Promotion Project of Chengdu University of TCM (BSH2019008), the Macao Science and Technology Development Fund (Macau Centre for Research and Development in Chinese Medicine, 007/2020/ALC), the Research Fund of University of Macau (CPG2022-00005-ICMS).

## Conflict of Interest

The authors declare that the research was conducted in the absence of any commercial or financial relationships that could be construed as a potential conflict of interest.

## Publisher’s Note

All claims expressed in this article are solely those of the authors and do not necessarily represent those of their affiliated organizations, or those of the publisher, the editors and the reviewers. Any product that may be evaluated in this article, or claim that may be made by its manufacturer, is not guaranteed or endorsed by the publisher.
